# Are Health Behavior Change Interventions That Use Online Social Networks Effective? A Systematic Review

**DOI:** 10.2196/jmir.2952

**Published:** 2014-02-14

**Authors:** Carol A Maher, Lucy K Lewis, Katia Ferrar, Simon Marshall, Ilse De Bourdeaudhuij, Corneel Vandelanotte

**Affiliations:** ^1^Health and Use of Time GroupUniversity of South AustraliaAdelaideAustralia; ^2^Exercise for Health and Human Performance GroupUniversity of South AustraliaAdelaideAustralia; ^3^School of MedicineUniversity of CaliforniaSan Diego, CAUnited States; ^4^Department of Movement and Sports SciencesUniversity of GhentGhentBelgium; ^5^Institute for Health and Social SciencesCentral Queensland UniversityNorth RockhamptonAustralia

**Keywords:** systematic review, social network, behavior change, intervention, Internet, physical activity, weight loss

## Abstract

**Background:**

The dramatic growth of Web 2.0 technologies and online social networks offers immense potential for the delivery of health behavior change campaigns. However, it is currently unclear how online social networks may best be harnessed to achieve health behavior change.

**Objective:**

The intent of the study was to systematically review the current level of evidence regarding the effectiveness of online social network health behavior interventions.

**Methods:**

Eight databases (Scopus, CINAHL, Medline, ProQuest, EMBASE, PsycINFO, Cochrane, Web of Science and Communication & Mass Media Complete) were searched from 2000 to present using a comprehensive search strategy. Study eligibility criteria were based on the PICOS format, where “population” included child or adult populations, including healthy and disease populations; “intervention” involved behavior change interventions targeting key modifiable health behaviors (tobacco and alcohol consumption, dietary intake, physical activity, and sedentary behavior) delivered either wholly or in part using online social networks; “comparator” was either a control group or within subject in the case of pre-post study designs; “outcomes” included health behavior change and closely related variables (such as theorized mediators of health behavior change, eg, self-efficacy); and “study design” included experimental studies reported in full-length peer-reviewed sources. Reports of intervention effectiveness were summarized and effect sizes (Cohen’s *d* and 95% confidence intervals) were calculated wherever possible. Attrition (percentage of people who completed the study), engagement (actual usage), and fidelity (actual usage/intended usage) with the social networking component of the interventions were scrutinized.

**Results:**

A total of 2040 studies were identified from the database searches following removal of duplicates, of which 10 met inclusion criteria. The studies involved a total of 113,988 participants (ranging from n=10 to n=107,907). Interventions included commercial online health social network websites (n=2), research health social network websites (n=3), and multi-component interventions delivered in part via pre-existing popular online social network websites (Facebook n=4 and Twitter n=1). Nine of the 10 included studies reported significant improvements in some aspect of health behavior change or outcomes related to behavior change. Effect sizes for behavior change ranged widely from −0.05 (95% CI 0.45-0.35) to 0.84 (95% CI 0.49-1.19), but in general were small in magnitude and statistically non-significant. Participant attrition ranged from 0-84%. Engagement and fidelity were relatively low, with most studies achieving 5-15% fidelity (with one exception, which achieved 105% fidelity).

**Conclusions:**

To date there is very modest evidence that interventions incorporating online social networks may be effective; however, this field of research is in its infancy. Further research is needed to determine how to maximize retention and engagement, whether behavior change can be sustained in the longer term, and to determine how to exploit online social networks to achieve mass dissemination. Specific recommendations for future research are provided.

## Introduction

Preventing and minimizing the impact of non-communicable diseases are some of the greatest challenges facing modern society. Key health behaviors, such as physical inactivity, smoking, obesity, poor diets, and alcohol misuse are among the most common causes of disease and premature deaths in both developed countries and, increasingly, developing countries [1-3]. For example, it has been estimated that approximately 2.6 million years of life are lost in England and Wales each year due to preventable disease burden [4]. Cost-effective, mass-reach public health interventions are needed to optimize health and well-being and minimize health care costs of lifestyle diseases.

A variety of media have been used to deliver mass-reach health campaigns, including television, radio, and billboard advertising [5], Web-based interventions [6], and recently, online social networks [7]. Online social networks have seen enormous growth in popularity in recent years and account for approximately one-quarter of all time spent online [8,9]. At present, there are many uncertainties as to whether, and how, online social networks might be harnessed to improve health: for example, how to handle privacy issues and whether people even desire to use online social networks to engage in health behavior change. Certainly, online social networks appear to offer considerable potential for delivery of public health campaigns, for several reasons. First, they can reach very large audiences (eg, Facebook, the world’s largest social networking website, has 1.1 billion users each month) [10]. Second, messages can be delivered via existing contacts, which may be more influential than health messages delivered via traditional marketing strategies [11]. Third, unlike traditional Web-based interventions [6], online social networks typically achieve high levels of user engagement and retention [10]. Finally, social media requires users to actively engage and generate content, which may well be more influential than traditional websites and advertising that are typically more passive in nature [12].

A number of studies have recently attempted to use online social networking strategies to instigate health behavior change. However, despite the large potential for behavior change and the immense popularity of online social networks, it is unclear how effective this approach has been across a range of different population groups and health behaviors. Therefore, this study aimed to systematically review the current level of evidence regarding the effectiveness of online social network health behavior interventions to influence tobacco and alcohol consumption, dietary intake, physical activity, and sedentary behavior.

## Methods

### Information Sources and Search Strategy

This review was undertaken and reported according to the Preferred Reporting Items for Systematic Reviews and Meta-Analyses (PRISMA) guidelines [13].

A preliminary search protocol was drafted and included terms for social media, the Internet, and the relevant health behaviors for this review. The search strategy was reviewed by experts in the area of online interventions (three members of the authorship team—CV, SM, and IDB) and an academic librarian before being finalized [14]. The final search was conducted on December 12, 2012 and included eight electronic databases: Scopus, CINAHL, Medline, ProQuest, EMBASE, PsycINFO, Cochrane, Web of Science and Communication & Mass Media Complete. Each database was searched individually and the search strategy for one database, Medline, is presented in Table 1. The search was limited to the English language, humans, and the year of publication from 2000 to present, and the search terms mapped to MeSH headings wherever possible. The reference lists of included studies and relevant systematic reviews were screened to identify further eligible studies.

**Table 1:** The search strategy as used in Medline.

Search category	Search terms
1. Social media	Social Networking/^a^ OR social network*.mp.^b^ OR social media.mp. OR Social Media/ OR (Facebook OR LinkedIn OR Twitter OR Badoo OR Orkut OR Qzone OR Xing OR Tencent OR Weibo OR Mixi OR Sina Weibo OR Hyves OR Skyrock OR Odnoklassniki OR Wer-kennt-wen OR V Kontakte OR Tuenti OR MySpace).mp.
2. Internet	online.mp. OR Internet/ OR internet.mp. OR web.mp.
3. Health behaviors	(cigarette.mp. OR Tobacco/ OR tobacco.mp. OR Smoking/ OR smoking.mp. OR Smoking Cessation/ OR nicotine.mp. OR Nicotine/) OR (alcohol.mp. OR Alcohol Drinking/ OR “binge drink*”.mp. OR “alcohol drink*”.mp.) OR (Motor Activity/ OR “physical activit*”.mp. OR “motor activit*”.mp. OR PA.mp. OR exercise.mp. OR Exercise/ OR exercis*.mp. OR sport*.mp. OR Sports/ OR MVPA.mp. OR Sedentary Lifestyle/ OR sedentar*.mp. OR sitting.mp. OR “screen time”.mp. OR inactiv*.mp. Television/ OR television.mp. OR TV.mp. OR Video games/ OR “video gam*”.mp.) OR (Diet/ OR diet*.mp. OR nutrition*.mp. OR “healthy eating”.mp. OR Food Habits/ OR Fruit/ OR fruit.mp. OR Vegetables/ OR vegetable*.mp. OR “snack food*”.mp. OR snack*.mp. OR” soft drink*”.mp. OR Carbonated beverages/) OR (Health Behavior/ OR “health behav*”.mp.)
4. Combined	1 AND 2 AND 3

^a^“/” denotes MeSH headings

^b^“.mp” denotes keyword search

### Study Selection

As per best practice for systematic reviews [15,16], eligibility of studies for inclusion in the review was determined by two independent reviewers (KF and either CM or LL), with results compared and disagreements discussed until consensus was reached. First, search results were screened based on the title and abstract and where eligibility was unclear or the abstract was unavailable the full text was obtained. The eligibility criteria were then applied to the full-text studies to determine inclusion in the review.

### Eligibility Criteria

#### Population

Adults or children were included, regardless of health status (healthy or participants with specific health conditions or diseases).

#### Intervention

Studies were included that reported an online intervention delivered either wholly or in part, using an online social network to deliver a health behavior change intervention. The online social network intervention could be delivered using an existing online social networking platform (eg, intervention delivered via either a “generic” pre-existing social networking website such as Facebook or Twitter, or a health-specific pre-existing social networking website, such as FatSecret) or a purpose-built intervention website incorporating social networking capabilities. In the case of purpose-built websites, studies had to explicitly describe their website as using social networking to be included. Interventions delivered via purpose-built websites which facilitated a degree of interactivity between participants (eg, a discussion board) but did not specifically describe the intervention as being or involving a “social network” were excluded.

#### Control or Comparator

Any comparator was acceptable (ie, a traditional control group, an alternative intervention, or a within subject pre-post design).

#### Outcomes

The online social media intervention had to target one of the following individual modifiable health behaviors identified by the World Health Organization as leading risk factors for global disease burden [3]: tobacco smoking, alcohol use, physical inactivity, or diet. For inclusion in the review, the study had to report data regarding the effectiveness of behavior change (eg, change in physical activity behavior [min/d]). Additionally, studies were included if they reported variables closely related to behavior change; this included potential mediators of behavior change (eg, dietary awareness or physical activity self-efficacy), or “downstream” variables (ie, variables that may have conceivably been impacted by health behavior change; eg, quality of life or body weight).

#### Study Design

Only experimental studies that were reported in peer-reviewed journals or as peer-reviewed full conference papers were included. Ecological studies, as well as studies employing small samples (eg, case studies), were eligible to be included in the review. Relevant systematic reviews were retained and the reference lists searched for additional relevant studies. Conference abstracts and theses were excluded.

### Data Collection Process and Data Items

Data extraction was conducted using a standardized form developed specifically for this review (see Multimedia Appendix 1), based upon that used by Davies et al [6]. For each included study, pairs of reviewers independently extracted data (CV/LL, CM/KF), with disagreements resolved by checking and discussing the original study until consensus was reached. Percent agreement between reviewers for data extraction was 88%, with the main discrepancies relating to classification of target behavior in the case of weight loss studies (whether the intervention targeted diet or weight loss or both). Extracted information included study participants (population, sample size, participation rate, attrition rate, recruitment method, setting), study design and duration of follow-up, behavior targeted, intervention description (including format, intensity, duration, and theoretical basis), and outcome measures used.

### Risk of Methodological Bias

The included studies varied widely in terms of research design, making selection of an appropriate risk of bias assessment tool difficult. After extensive discussion among the research team, a tool was devised based upon the CONSORT checklist [17]. The tool comprised 25 items, with items scored as 1 or 0 if the studies satisfactorily met/didn’t meet the criteria (see Multimedia Appendix 2), with a higher score indicating lower risk of methodological bias. While intended for controlled trials, the team felt that the majority of items (20 out of 25) were applicable to other study designs and that the weaker study designs rightly should receive a lower score than studies utilizing a controlled trial design. Each study was also ranked using the 2011 Centre for Evidence Based Medicine Levels of Evidence, where Level 1 signifies systematic review of randomized trials; Level 2 randomized trial or observational study with dramatic effect; Level 3 non-randomized controlled cohort/follow-up study; Level 4 case-series, case-control studies, or historically controlled studies; and Level 5 mechanism-based reasoning [18]. Rating of studies was conducted independently by pairs of reviewers (CM/LL, CV/KF) with any differences resolved by discussion. Percent agreement between reviewers for the scoring of risk of methodological bias was 81%, with the most common points of discrepancy relating to whether the trial was registered and whether a trial protocol was accessible.

### Summary Measures and Synthesis of Results

The primary outcome measure was health behavior change (for example, physical activity and dietary behaviors). Secondary outcome measures related to behavior change were also examined. These could be either “downstream” from behavior change (ie, outcomes brought about by sustained behavior change, for example, change in body weight) or “upstream” (ie, theorized mediators of behavior change, such as knowledge, attitudes, or self-efficacy).

To determine whether the interventions had a significant impact on behavior, we evaluated and coded individual outcomes. In studies without a control group, a positive outcome was recorded if there was a statistically significant change across time. In the case of controlled trials, studies were coded as having a positive outcome if statistically significant differences between groups across time were reported. In the case of controlled trials where the intervention was compared with an alternative intervention (as opposed to a no-intervention control group) and there was a significant improvement in both groups, but not between groups, this was coded as a “suggested positive” outcome. To allow for comparison across studies, effect sizes (Cohen’s *d* and 95% confidence intervals) were calculated according to formulas published by Lipsey and Wilson [19] (online calculators available at [20]). The magnitude of the effect sizes were classified using descriptors proposed by Thalheimer and Cook, where effect sizes ≥−0.15 and <0.15 are “negligible”, ≥0.15 and <0.40 are “small”, ≥0.40 and <0.75 are “medium”, ≥0.75 and <1.10 are “large”, ≥1.10 and <1.45 are “very large”, and ≥1.45 are “huge” [21].

Attrition, engagement, and fidelity with the social networking component of the interventions were scrutinized where sufficient data were presented to allow this. Fidelity was calculated by comparing the actual engagement with the intended dosage.

## Results

### Study Selection

**Figure 1:**
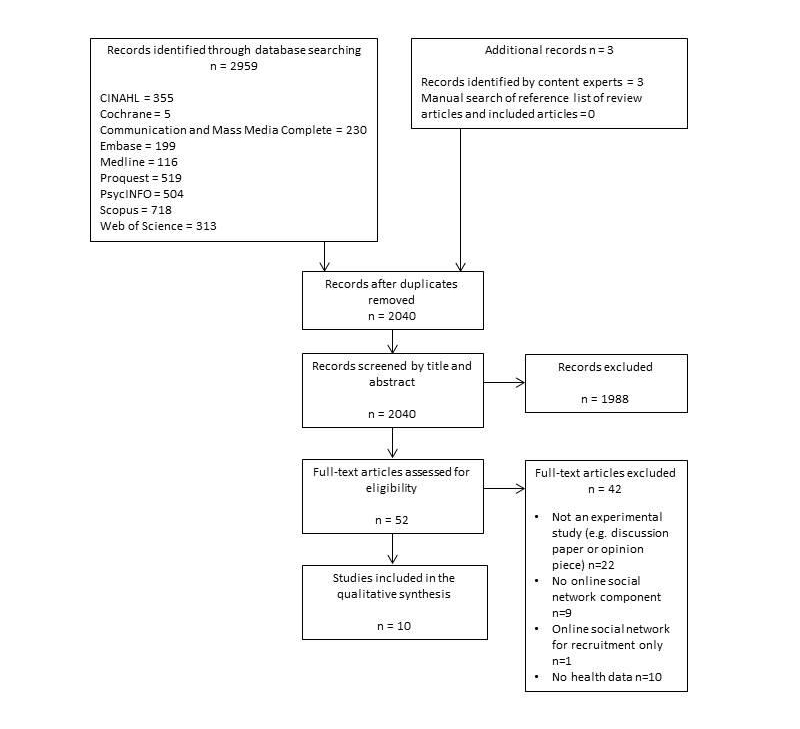
Flow of studies through the review.

A total of 2040 studies were identified from the database search following removal of duplicates. The flow of studies through the review is summarized in Figure 1. Ten articles reporting data regarding the effectiveness of online social networking behavior change interventions were included in the review.

### Study Characteristics

A summary of the key characteristics of included studies is presented in Multimedia Appendix 3. The total number of participants across the 10 studies was 113,988. The studies typically reported high rates of female participation: on average 83.3% of participants were female. The targeted health behaviors were diet/weight loss (n=2), physical activity (n=3), or a combination of diet/weight loss and physical activity (n=5). No eligible studies targeted smoking or alcohol consumption. Five studies involved interventions delivered solely via online social networks [22-26] and the remaining studies involved interventions that used online social networks in conjunction with other intervention strategies [27-31], including standalone intervention websites, printed materials, and provision of supplemental equipment such as kitchen scales and utensils. Only three interventions were reported to be theoretically-based; of these, Social Cognitive Theory was reported in two studies [27,28], and Social Learning Theory [24] was reported in a single study. One further study used a behavior change theory during evaluation (the Theory of Planned Behavior), but not during intervention development [22]. All of the interventions facilitated or encouraged daily use. Interventions ranged in duration from 5 days to 6 months. No studies reported a long-term follow-up to determine whether outcomes were sustained beyond the intervention period.

### Study Methodology

Three key study types were identified: (1) large-scale evaluations of “live” interventions, typically with >1000 participants (four studies with sample sizes ranging from 545 to 107,907) [22,24-26], (2) medium-scale, tightly-controlled randomized controlled trials, typically with approximately 100 participants (four studies with sample sizes ranging from 52 to 134) [27-29,31], and (3) small pilot studies, each with 10 participants (two studies) [23,30]. In all, five studies were randomized controlled trials (RCT) [22,27-29,31], one was a randomized cross-over study [23], and four were single group pre-post studies [24-26,30]. Of the six studies that utilized a separate control group or arm (crossover study), only one had a “true” (ie, no-intervention) control [29], with the others comparing the online social networking intervention with an alternative intervention (in five cases the alternative intervention was Web-based [22,23,27,28,31], and in three cases the alternative intervention involved an online social networking component [22,23,27].

### Recruitment Methods and Rates

Eight of the 10 included studies recruited participants to their interventions (the other two were evaluations based upon existing users of commercial online social networks [25,26]). Of these, six described their recruitment strategies, with all of these studies reporting a variety of traditional recruitment methods, such as advertising with flyers [27,29,31], mainstream media [22,28], and mass emails [27-29,31]. Only one study [27] reported using an online social media campaign, in addition to other recruitment methods. Participation rates varied widely, ranging from 33% [22] to 89% [27]. However, it is important to note that participation rates were only reported relative to the total number of volunteers coming forward and not the total number of people exposed to recruitment materials.

### Risk of Methodological Bias

Risk of methodological bias scores ranged from high (19.5 out of 25) [28], to low (0.5 out of 25) [26]; for full details, see Multimedia Appendix 2. In general, the large-scale “live” interventions scored poorly on the risk of bias assessment (range 0.5-4 out of 25, with the exception of Brindal et al [22], which scored 16.5 out of 25), the medium-scale RCTs scored highest (range 11-19.5 out of 25), and the pilot studies scored poorly (range 4-8.5 out of 25).

Most studies met the CONSORT requirements to provide a strong scientific rationale and described their interventions clearly. However, none of the studies met the stringent guidelines for quality reporting of trial results, which requires provision of effect size estimates and their precision. Only one study reported that participants were blinded to the treatment condition [22]. Attrition rates were reported by eight studies, while participation rates were reported in only five studies.

### Intervention and Follow-Up Duration

Interventions ranged from 5 days [23] to 6 months in duration [28]. No studies reported follow-up of outcomes and maintenance of behavior change beyond the end of the intervention itself.

### Efficacy

Four studies (three pre-post studies and one cross-over study) reported significant improvement in an outcome measure, namely weight loss (n=2) [25,26], physical activity (n=1) [23], and dietary awareness (n=1) [30]. A further four studies, all randomized controlled trials employing alternative intervention controls, reported evidence suggestive of improvement (ie, both groups improved significantly over time, though there was no significant difference between groups) [22,27,28,31]. The remaining two studies [24,29] reported mixed findings; for example, Napolitano et al’s randomized controlled trial reported significant weight loss in the Facebook Plus group relative to controls over time, but not the Facebook group relative to controls [29] (Multimedia Appendix 3).

**Figure 2:**
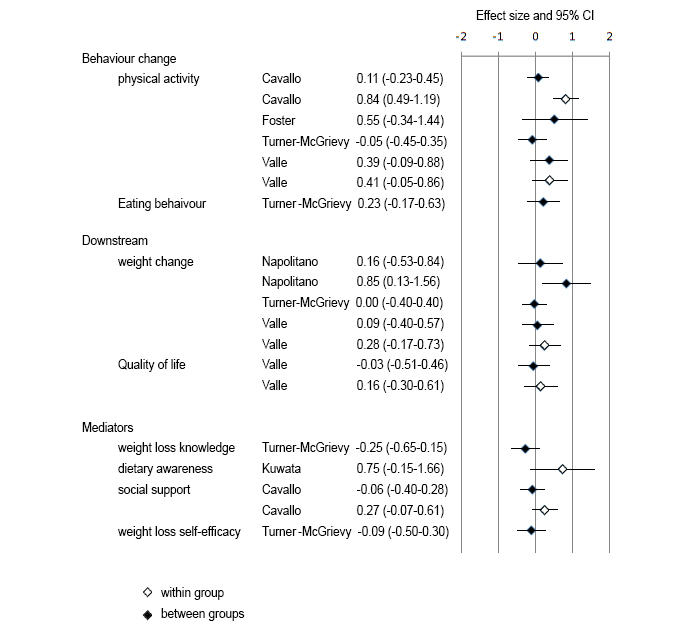
Forest plot of effect sizes for behavior change, downstream, and mediator variables.

Meta-analysis was not completed due to the relatively small number of studies included and the wide variety of interventions, comparators, and study designs employed in the studies. However, effect sizes were calculated for the six studies that provided sufficient data to do so (Figure 2). One RCT [29] employed a “true” (ie, no intervention) control; therefore, between group differences are presented. The other three RCTs providing sufficient data to calculate effect sizes used alternative interventions as their control condition [27,28,31]. Therefore, wherever possible, both between group and within group effect sizes (based on pre and post data for the intervention group only) were calculated for these studies. Despite numerous studies reporting statistically significant changes (refer to Table 2), few studies returned significant effects when the 95% confidence intervals were calculated. The magnitude of the effect sizes are summarized below.

**Table 2:** Summary of intervention effects on behavior, downstream, and mediator outcome measures.^a^

Study	Behavior outcomes			Downstream outcomes		Mediators						
	PA^b^	Energy intake	Eating behavior	Weight loss	QOL^c^	Weight loss knowledge	Dietary awareness	PA awareness	Health attitudes	PA self-efficacy	Weight self-efficacy	Social support
Brindal et al [22]				+								
Cavallo et al [31]	+											±
Foster et al [23]	++											
Freyne et al [24]									±*			
Kuwata et al [30]							++	−				
Ma et al [25]				++								
Napolitano et al [29]	−			±*						−	−	−
Sugano & Yamazaki [26]				++								
Turner-McGrievy & Tate [28]	(+)	(+)	(+)	(+)		(+)					(+)	
Valle et al [27]	+			+	±*							

^a^+: within-group significant improvements, in RCT with alternative intervention; ±: within-group mixed results; some significant improvements, some no change, in RCT with alternative intervention; ++: significant improvement; −: no significant change; ±*: mixed results; some subscales showed significant improvement, some showed no significant change; (+) within-group improvements but significance not reported, in RCT with alternative intervention.

^b^PA: physical activity

^c^QOL: quality of life

### Behavior Change

Of the four studies that investigated physical activity behavior change and reported sufficient data to enable effect size calculation, the effect size in one study was classified as negligible [28], two as medium (between groups) [23,27], and one as large (between groups) [31]. A small effect size was observed for the one study measuring eating behavior [28].

### Downstream Variables

Three studies measured weight change and reported sufficient data to permit effect size calculation. Effect sizes ranged from negligible [28], to small [27], to large [29] (Facebook Plus versus control). One study measured quality of life and reported negligible to small effects [27].

### Mediators

A small negative effect size was calculated for the single study that measured weight loss knowledge, though it is worth noting this study employed a substantial alternative intervention control [28]. The same study showed a negligible effect for weight loss self-efficacy. A single study found a negligible to small effect for social support [31], and one study showed a large effect for dietary awareness [30].

### Attrition, Engagement, and Fidelity

Attrition rates (ie, participant dropout over the course of the study) varied by study design, with the small scale pilot studies reporting the lowest attrition (0%), the mid-sized RCTs reporting low attrition (4 [29] -23% [27]) and the large live trials reporting high attrition (ranging from 41% in [25] to 84% in [22]; note that attrition rates were not reported in [24] and [26]). Where possible, we examined engagement with the social networking component of the intervention in each study and compared it with the intended dosage, to provide an indication of fidelity. Results (Table 3) showed that fidelity was generally quite low. With the exception of Foster et al [23], which achieved higher usage rates than intended (105%), the other studies reporting usage rates were only 5 to 15% of that intended.

All three studies that reported how engagement changed over the course of the intervention found that it gradually declined [24,27,28]. Studies that compared a social intervention with a non-social control reported that the social intervention achieved higher engagement [22-24] and had higher user satisfaction [22] than the non-social intervention. Weight loss was significantly associated with engagement [22,26] in the two studies that reported this subgroup analysis. Sugano and Yamazaki [26] also reported that extent of social interaction was positively associated with weight loss.

**Table 3:** Summary of engagement and fidelity with the interventions.

		Usage rates	
Study	Intended number of uses	Actual usage (=“engagement”)	% intended: actual (=“fidelity”)
Brindal et al [22]	84	6.0	7.1%
Cavallo et al [31]	84	5.1	6.1%
Foster et al [23]	21	22	104.8%
Ma et al [25]	133	6.1	4.6%
Turner-McGrievy & Tate [28]	364	54.6	15.0%
Valle et al [27]	84	4.6	5.5%

## Discussion

### Principal Findings

This systematic review found modest evidence that online social network interventions may be effective, with 9 of the 10 included studies reporting significant improvements in some aspect of health behavior or related outcomes. However, effect sizes for behavior change were generally small.

This review identified that online social network-based interventions to date have taken one of two key approaches: (1) some have developed interventions that used popular existing online social networking websites, such as Facebook and Twitter, and (2) others have developed standalone, health-focused online social networks. Results suggest that standalone health-focused online social networks can be effective for the users they retain over a period of time; however, poor retention is an issue, with roughly 50% or more of users who sign up failing to stay in the intervention for its duration, and for those who do, engagement is generally low. It may also be argued that a drawback of health-focused online social networks is that they are likely to attract motivated individuals who were already contemplating changing their health behavior.

Using popular existing social network sites may address issues of reach, engagement, and retention. For example, Facebook reports that 61% of its total users log in daily [32]. Certainly, the studies included in this review [23,27,29,31] that used Facebook managed to retain a high proportion of participants across the study period (77-96% of users). However, they typically did not achieve high engagement (5-15%) [27,29,31], with the exception of the Foster study, which achieved 105% of intended use [23]. That engagement was typically low is concerning, given that these studies utilized extensive participant contact, prompting, and email, which are likely to have inflated engagement compared to what might be seen in a more ecologically valid setting. The intervention approach used in the Foster study [23] was considerably different to that used in the other Facebook studies [27,29,31], which might explain the different levels of engagement observed. Foster [23] recruited participants who already knew each other and created a friendly competitive environment with a tally board. In contrast, the other studies have tended to use Facebook and Twitter as a social support tool, where intervention participants (who were strangers to each other) were encouraged to share information and advice [27,29,31]. It could be argued that the approach used by Foster [23] was more in tune with how people use online social networks, given that people more commonly use Facebook to interact with people with whom they share an offline connection as well, rather than using Facebook to interact with new people [33]. Furthermore, entertainment is recognized as being a key motivator for Facebook use [34]; the friendly-competitive tone of the Foster intervention was probably consistent with this. The Foster study only ran for 21 days, considerably less than the other interventions, which each lasted 12 weeks. It seems unlikely that the very high engagement achieved by Foster et al [23] would have been sustained over a longer duration. Despite this, the high engagement achieved in those 3 weeks suggests this friendly-competitive intervention may be a promising approach.

### Strengths and Limitations

Strengths of this systematic review are that it was conducted and reported according to PRISMA guidelines [13]. It utilized a rigorous and comprehensive search strategy. Study selection, data extraction, and critical appraisal were completed in duplicate by two members of the research team independently, ensuring the accuracy of the review data.

A key limitation of the review was the heterogeneity of the identified studies. Studies varied in terms of target population, intervention, and study design. Furthermore, only a relatively small number of eligible studies were identified. A large number of academic databases were searched (eight), and an academic librarian was consulted regarding which databases should be used; however, it is always possible that other databases may have uncovered additional studies. These factors limited our ability to synthesize data and reach definitive conclusions. It is also important to note that the included studies varied widely in terms of risk of bias, with some studies scoring very poorly, which reduces the trust that can be placed in their findings. Finally, the possibility of publication bias should also be acknowledged. As with all systematic reviews examining the efficacy of interventions, there is a possibility that studies with null findings have not been published [16], and that the synthesis of data presented here gives an overly favorable account of effectiveness.

### Future Research

This review offers preliminary evidence that social networking-based health interventions may be effective in changing behavior. However, this field of research is in its infancy and many questions remain unanswered. It is currently unclear whether social networking-based interventions are equally useful for all health behaviors or whether they may be more effective for some than others. The identified studies only followed participants for a relatively short period (the longest was 6 months). Given that many of the health benefits of health behavior are achieved over a long-term period, further work is needed to examine whether the short-term behavior change achieved in the included studies can be sustained over a longer period, such as 12 months and beyond [35]. It will also be important to determine whether sustained interaction with the user interface is required to sustain behavior change or whether behavior change may persist after interaction with intervention materials ceases. Delivery of interventions that use existing online social networks such as Facebook and Twitter appear to offer particular promise for sustained engagement, due to their high level of user retention and engagement, whether retention and engagement with specific aspects of these platforms (such as a specific app or Facebook group delivering a health intervention) matches this is currently unclear. Innovative approaches reflecting the way people use online social networks (with existing friends and for entertainment) are warranted. In particular, gamification (ie, the use of video gaming elements such as collecting virtual points or badges) in non-gaming situations is an emerging trend in online campaigns and offers promise for improving user experience and engagement [36].

Interestingly, to date, the interventions that have used existing popular online social networks have still used traditional methods of recruitment (eg, flyers, media advertising), and have also been highly controlled (eg, group membership has been closed in order to prevent contamination between study groups). This contrasts with the touted benefits of using online social networks for health intervention, such as the ability to recruit participants via social networks [37], and to virally disseminate interventions on a mass scale [38]. There is clearly a role for tightly controlled randomized controlled trials in order to establish efficacy of an intervention approach; however, ecological study designs, which closely mimic real conditions of social network use, are also required in order to learn how to best exploit viral properties of online social networks for mass dissemination [7]. Cross-disciplinary research pairing health behavior change experts with social marketers may help determine how to most effectively use online social networks for recruitment and mass dissemination.

### Recommendations for Future Studies

More studies are needed that attempt to intervene in health behavior using online social networks. The following recommendations for future research may be useful for both health researchers and human-computer interaction researchers who design and implement technology-based interventions integrating social networking to facilitate health interventions:

1. Design social-networking interventions that can be delivered primarily within the social network setting. Provision of a small degree of supplementary equipment or printed resources is reasonable, but it is important to recognize that interventions incorporating multiple physical resources have limited ecological validity.
2. Examine interventions delivered via existing popular social network websites, such as Facebook, given their proven ability to attract and retain participants and potential for mass dissemination. Such interventions should be responsive to the way people use online social networks (predominantly with existing friends and for entertainment).
3. Utilize large sample sizes to ensure they are sufficiently powered to detect effects, should they exist.
4. Involve high quality research methods, such as carefully designed randomized controlled trials.
5. In addition to high-quality efficacy studies, ecologically valid studies such as pragmatic randomized trials are also required to determine interventions’ abilities to mass disseminate in a real-world setting.
6. Emphasize online recruitment strategies.
7. Involve long-term follow-up (eg, behavior change at 12 months and beyond).

### Conclusion

In conclusion, research using online social networks to bring about health behavior change is still in its early stages of development and, while several studies show promise, much is still to be learned about optimizing these interventions to increase their efficacy. In particular, research is needed to determine how to maximize retention and engagement, whether behavior change can be sustained in the longer term, and to determine how to exploit online social networks to achieve mass dissemination.

## Multimedia Appendix 1

Data extraction form.

## Multimedia Appendix 2

The Risk of Bias tool, based upon the CONSORT checklist.

## Multimedia Appendix 3

Overview of the characteristics and outcomes of the included studies.

## Abbreviations

PA: physical activity
PRISMA: Preferred Reporting Items for Systematic Reviews and Meta-Analyses
QOL: quality of life
RCT: randomized controlled trial
