# Supplementary material for: Are Health Behavior Change Interventions That Use Online Social Networks Effective? A Systematic Review
Source: J Med Internet Res. 2014 Feb 14;16(2):e40. doi: 10.2196/jmir.2952 (PMC3936265; doi:10.2196/jmir.2952)
Supplement: Supplementary file 1 [file jmir_v16i2e40_app1.pdf]

Multimedia Appendix 1. The data extraction form.

	Characteristic	Codes
1	Publication status	1. Published<br>2. In press/accepted for publication
2	Design of study	1. Randomised controlled trial<br>2. Pre-post measures<br>3. Within subject 2 conditions
3	Participant selection	1. Convenience sample of non-random participants<br>2. Random selection<br>3. Representative sample<br>4. Other
4	Behaviours targeted in intervention	1. Physical activity<br>2. Diet<br>3. Weight loss<br>4. Smoking cessation<br>5. Alcohol consumption
5	Sample size at commencement of study	
6	Average age of sample	
7	% female participants	
8	% population white	
9	Health status of target population	1. Diabetes<br>2. Cancer<br>3. Absence of chronic disease<br>4. Overweight<br>5. General<br>6. Not specified<br>7. Metabolic syndrome<br>8. Physical disabilities<br>9. Chronic Disease<br>10. Arthritis<br>11. Chronic Obstructive Pulmonary Disease<br>12. Cardiovascular disease
10	Screening based on target behaviour	1. Sedentary population (not meeting physical activity guidelines)<br>2. Overweight<br>3. Other
11	Country of origin	1. USA<br>2. Australia<br>3. England<br>4. Singapore<br>5. New Zealand<br>6. Canada<br>7. Belgium<br>8. Norway<br>9. Taiwan<br>10. South Korea<br>11. Netherlands
12	Study setting	1. University<br>2. Hospital staff<br>3. School<br>4. Other (specify)
13	Type of measures used	1. Self-report<br>2. Objective
14	Self-report physical activity methods used	1. IPAQ (short form)<br>2. IPAQ (long form)<br>3. BRFSS<br>4. 2D PAR

		5. NHANES III<br>6. Active Australia<br>7. Godin<br>8. 7-day recall<br>9. Not required<br>10. Other
15	Reliability of self-report physical activity measure reported	1. Yes<br>2. No<br>3. Not reported
16	Validity of self-report physical activity measure reported	1. Yes<br>2. No<br>3. Not reported
17	Objective physical activity measure	1. Accelerometer<br>2. Pedometer<br>3. Direct observation<br>4. Not required
18	Physical activity mode assessed	1. Overall physical activity<br>2. Walking<br>3. Leisure time physical activity<br>4. Vigorous intensity physical activity<br>5. Moderate intensity physical activity<br>6. Light intensity physical activity<br>7. Other
19	Other outcome measured 1 (write descriptively)	
20	Name of tool used 1 (write descriptively)	
21	Reliability and/or validity of measurement tool reported 1	1. Yes<br>2. No<br>3. Not reported
22	Other outcome measured 2 (write descriptively)	
23	Name of tool used 2 (write descriptively)	
24	Reliability and/or validity of measurement tool reported 2	1. Yes<br>2. No<br>3. Not reported
25	Other outcome measured 3 (write descriptively)	
26	Name of tool used 3 (write descriptively)	
27	Reliability and/or validity of measurement tool reported 3	1. Yes<br>2. No<br>3. Not reported
28	Number of groups in study	
29	Type of baseline measure	1. Face-to-face<br>2. Telephone<br>3. Internet<br>4. Regular mail
30	Type of introduction session to intervention	1. Face-to-face<br>2. Telephone<br>3. Internet<br>4. Not specified
31	Duration of intervention (weeks)	
32	Number of follow up time points (other than immediately post intervention)	
33	Length of time from post-test measure to first follow up (weeks)	
34	Length of time from post-test measure to second follow up (weeks)	
35	Length of time from post-test measure to third follow up (weeks)	
36	Number of contacts throughout the intervention (other than website)	

37	Number of contacts throughout the intervention over the internet (intervention group only)	
38	Additional contacts throughout the intervention face-to-face? (intervention group only)	1. Yes<br>2. No
39	Additional contacts throughout the intervention over the phone? (intervention group only)	1. Yes<br>2. No
40	Additional contacts throughout the intervention via print based materials? (intervention group only)	1. Yes<br>2. No
41	Intervention tailored to individual	1. Comprehensive (major component)<br>2. Limited (part of intervention tailored)<br>3. No tailoring
42	Intervention developed based on theory?	1. Yes<br>2. No
43	Which theory	1. Social cognitive theory<br>2. Theory of planned behaviour<br>3. Transtheoretical model (incl. Stages of change)<br>4. Stages of change (not include Transtheoretical model)<br>5. Self-management theory<br>6. Social ecological model<br>7. Protection motivation theory<br>8. Goal-setting theory<br>9. Health beliefs model<br>10. Other (write out)
44	Was the OSN the sole intervention, or only a component of a larger intervention?	1. OSN only<br>2. Standalone website plus OSN<br>3. Other (describe)
45	Education included in **OSN component** of the intervention (structured education material)	1. Yes<br>2. No
46	Feedback included in **OSN component** of the intervention (any form of feedback)	1. Yes<br>2. No
47	Goal setting included in **OSN component** of the intervention	1. Yes<br>2. No
48	Online self-monitoring for target behaviour included in **OSN component** of the intervention	1. Yes<br>2. No
49	Email reminders included in **OSN component** of the intervention	1. Yes<br>2. No
50	Updated content included in **OSN component** of the intervention	1. Yes<br>2. No
51	Discussion board/forum communication included in **OSN component** of the intervention (asynchronous communication - e.g. discussion board where you aren't expecting a response straight away)	1. Yes<br>2. No
52	Chat communication included in **OSN component o**f the intervention (synchronous communication - i.e. sending chat messages with reply in real time)	1. Yes<br>2. No
53	Quiz included in **OSN component** of the intervention	1. Yes<br>2. No
54	Any type of facilitator or moderator function included in **OSN component** of the intervention	1. Yes<br>2. No
55	Education included in **Non OSN component** of the intervention (structured education material)	1. Yes<br>2. No
56	Feedback included in **Non OSN component** of the intervention (any form of feedback)	1. Yes<br>2. No
57	Goal setting included in **Non OSN component** of the intervention	1. Yes<br>2. No

58	Online self-monitoring for target behaviour included in **Non OSN component** of the intervention	1. Yes<br>2. No
59	Email reminders included in **Non OSN component** of the intervention	1. Yes<br>2. No
60	Updated content included in **Non OSN component** of the intervention	1. Yes<br>2. No
61	Discussion board/forum communication included in **Non OSN component** of the intervention (asynchronous communication - e.g. discussion board where you aren't expecting a response straight away)	1. Yes<br>2. No
62	Chat communication included in **Non OSN component o**f the intervention (synchronous communication - i.e. sending chat messages with reply in real time)	1. Yes<br>2. No
63	Quiz included in **Non OSN component** of the intervention	1. Yes<br>2. No
64	Any type of facilitator or moderator function included in **Non OSN component** of the intervention	1. Yes<br>2. No
65	Percent of attrition throughout study for all groups	
66	Percent of attrition throughout study for intervention group only	
67	Participation rate of study	
68	Website usage of participants throughout intervention (average logins per participant)	
69	Type of control group used	1. True (e.g. wait list)<br>2. Standard care<br>3. Minimal OSN intervention<br>4. Minimal non-OSN intervention<br>5. Alternative intervention<br>6. Other (write out)
70	Outcome 1 (name it) Any significant improvements in outcome measured in intervention group?	1. Yes - significant improvement compared with control<br>2. "Yes perhaps" if both groups improved, in the case of minimal OSN control or alternative intervention control<br>3. No
71	Outcome 2 (name it) Any significant improvements in outcome measured in intervention group?	1. Yes - significant improvement compared with control<br>2. "Yes perhaps" if both groups improved, in the case of minimal OSN control or alternative intervention control<br>3. No
72	Outcome 3 (name it) Any significant improvements in outcome measured in intervention group?	1. Yes - significant improvement compared with control<br>2. "Yes perhaps" if both groups improved, in the case of minimal OSN control or alternative intervention control<br>3. No
73	Is enough info presented that we would be able to calculate an effect size for behaviour change (sample size, group means and standard deviations reported)	1. Yes<br>2. No

OSN = online social network
