# Supplementary material for: Are Health Behavior Change Interventions That Use Online Social Networks Effective? A Systematic Review
Source: J Med Internet Res. 2014 Feb 14;16(2):e40. doi: 10.2196/jmir.2952 (PMC3936265; doi:10.2196/jmir.2952)
Supplement: Supplementary file 2 [file jmir_v16i2e40_app2.pdf]

Multimedia Appendix 2. The Risk of Bias tool, based upon the CONSORT checklist.

	Cavallo	Freyne	Ma	Napolitano	Sugano	Turner	Valle
**1. Title and abstract:**	✓ ✓	- -	- -	- ✓	- -	✓ ✓	✓ ✓
a) identification as a randomised trial in title; b) structured summary							
**2. Introduction**	✓ ✓	✓ ✓	✓ -	✓ ✓	- -	✓ -	✓ ✓
a) scientific background/ rationale; b) specific objectives/ hypotheses							
**3. Methods – *trial design***	✓ -	- -	- -	✓ -	- -	✓ -	✓ -
a) description of trial design; b) changes to methods after trial commencement							
4. Participants	✓ ✓	- ✓	- -	✓ ✓	✓ -	✓ ✓	✓ ✓
a) eligibility criteria; b) settings and locations of data collection							
5. Interventions	✓	-	✓	✓	-	✓	✓
Descriptions with sufficient details to allow replication							
6. Outcomes	- -	- -	- -	✓ -	- -	✓ -	✓ -
a) pre-specified primary and secondary outcome measures; b) changes to outcomes after trial commenced							
7. Sample size	✓	-	-	-	-	✓	✓ -

a) how sample size was determined; b) if applicable, interim analyses/ stopping guidelines							
**8. Randomisation – *sequence generation***	- -	- -	- -	- -	-	✓ ✓	✓ ✓
a) method used; b) type of randomisation including details of any restriction							
9. Allocation concealment mechanism	-	-	-	-	-	✓	-
Implementation of the random allocation sequence, including concealment							
10. Implementation	-	-	-	-	-	✓	-
Who generated the random allocation sequence, who enrolled participants, and who assigned participants							
11. Blinding	-	-	-	-	-	-	-
a) if done, who was blinded and how; b) if relevant, similarity of interventions							
12. Statistical methods	✓ -	- -	- ✓	✓ ✓	- -	✓ ✓	✓ ✓
Statistical methods used a) for primary and secondary outcomes; b) additional analyses							
**13. Results – *participant flow***	✓ ✓	- -	✓ -	✓ ✓	- -	✓ ✓	✓ ✓

a) numbers of participants randomised, receiving treatment, and analysed; b) losses and exclusions, with reasons							
14. Recruitment	✓ -	✓ -	✓ -	✓ -	- -	✓ ✓	✓ -
a) dates of recruitment and follow-up; b) why the trial ended							
15. Baseline data	-	-	-	-	-	✓	✓
A table with baseline demographic and clinical characteristics for each group							
16. Numbers analysed	✓	-	-	-	-	✓	✓
For each group, number of participants included in each analysis							
17. Outcomes and estimation	- -	- -	- -	- -	- -	- -	- -
a) results for each group, and the estimated effect size and its precision; b) absolute and relative effect sizes for binary outcomes							
18. Ancillary analyses	-	-	-	✓	-	✓	✓
Results of any other analyses performed, distinguishing pre-specified from exploratory							
19. Harms	-	-	-	-	-	-	✓
Harms or unintended effects in each group							

**20. Discussion** - ***Limitations***	✓	-	-	✓	-	✓	✓
Trial limitations/bias/ multiplicity of analyses							
21. Generalisability	-	-	-	-	-	✓	✓
Generalisability (external validity, applicability) of findings							
22. Interpretation	✓	-	✓	✓	-	✓	✓
Consistent with results and balanced							
**23. Other information –** ***Registration***	✓	-	-	-	-	✓	✓
Registration number and name of registry							
24. Protocol	✓	-	-	-	-	✓	✓
Where the full trial protocol can be accessed							
25. Funding	✓	✓	-	✓	-	✓	✓
Sources of funding/ role of funders							
Number of criteria satisfied	13.5	3	4	11	0.5	19.5	18
