# Supplementary material for: Are Health Behavior Change Interventions That Use Online Social Networks Effective? A Systematic Review
Source: J Med Internet Res. 2014 Feb 14;16(2):e40. doi: 10.2196/jmir.2952 (PMC3936265; doi:10.2196/jmir.2952)
Supplement: Supplementary file 3 [file jmir_v16i2e40_app3.pdf]

Study	Risk of bias (/ 25) and CEBM level of evidence	Design/ participants/ setting	Intervention	Online social-networking features	Outcome measures	Process measures
Brindal et al, 2012 [21]	CONSORT<br><br>= 16.5;<br><br>CEBM Level 2	**Participants**: 2648 overweight volunteers.<br><br>Age: mean 45.0 (SD 11.9)<br><br>**% female**: 83.4<br><br>**Participation rate**: 33%<br><br>**Attrition rate**: 84%<br><br>**Recruitment**: mass media campaign<br><br>**Setting**: live trial<br><br>**Country**: Australia<br><br>**Design**: RCT	**Focus**: diet & weight loss<br><br>**Groups**: (a) personalised-online social network including personalised tools; (b) online social network including non-personalised tools; (c) information-based website.<br><br>**Additional to online social-network**: 7 emails<br><br>**Duration**: 12 weeks<br><br>**Follow-up post baseline**: 12 weeks<br><br>**Intro session**: none	**Format:** Health online social networking website ("Online CSIRO Total Wellbeing Diet")<br><br>**Frequency:** daily use encouraged (i.e. total 84 logins).<br><br>**Characteristics**: education, feedback, self-monitoring, email reminders (n=7), updated content, discussion board/forum, quiz, facilitator/moderator<br><br>**Theory**: Theory of Planned Behaviour	**Primary outcome:** Self-reported weight and BMI.<br><br>**Tool:** NA<br><br>**Validity:** NR<br><br>**Secondary outcomes:** Numerous mediators reportedly measured (body dissatisfaction, proactive coping, perceived need to lose weight, behavioural intention), but results not presented.	Mean logins varied significantly between groups: 6.0 logins for the personalised-supportive group; 5.5 for the supportive group and 3.4 for the control group, over the 12 week intervention.<br><br>Satisfaction with website: significantly higher for online social network sites compared with control.<br><br>Usefulness of website: significantly higher for online social network sites compared with control.
Cavallo et al, 2012 [30]	CONSORT<br><br>= 13.5;<br><br>CEBM Level 2	**Participants**: 134 healthy, physically inactive college students<br><br>**Age**: mean NR. <25 years<br><br>**% female**: 100<br><br>**Participation rate**: 64%<br><br>**Attrition rate**: 10%<br><br>**Recruitment**: advertising within	**Focus**: physical activity<br><br>**Groups**: (a) physical activity website including self-monitoring features plus Facebook group; (b) physical activity website and emails.<br><br>**Additional to online social-network**: >38	**Format**: Facebook group, to supplement a physical activity website<br><br>**Frequency:** daily use encouraged (i.e. total 84 logins).<br><br>**Characteristics:** education, feedback from moderator, email reminders (n>38), updated content, discussion board/forum,	**Primary outcome:** Self-reported physical activity.<br><br>**Tool:** Paffenbarger activity questionnaire<br><br>**Validity:** NR<br><br>**Secondary outcomes:** Online social support for physical activity	Mean logins to standalone website = 8.7 logins per participant; mean logins to Facebook = 5.1 over 12 weeks.

Study	Risk of bias (/ 25) and CEBM level of evidence	Design/ participants/ setting	Intervention	Online social-networking features	Outcome measures	Process measures
		university<br><br>**Setting**: university students<br><br>**Country**: United States<br><br>**Design**: RCT	emails.<br><br>**Duration**: 12 weeks<br><br>**Follow-up post baseline**: 12 weeks<br><br>**Intro session**: none	facilitator/moderator<br><br>**Theory:** NR		
Foster et al, 2010 [22]	CONSORT<br><br>= 8.5;<br><br>CEBM Level 2	**Participants**: 10 healthy adults<br><br>**Age**: NR<br><br>**% female**: 90<br><br>**Participation rate**: NR<br><br>**Attrition rate**: 0%<br><br>**Recruitment**: personal contacts<br><br>**Setting**: hospital nursing staff<br><br>**Country**: United Kingdom<br><br>**Design**: randomised cross-over study	**Focus**: physical activity<br><br>**Groups**: (a) Facebook app with self-monitoring and online social interaction (2) Facebook app with self-monitoring only.<br><br>**Additional to online social network**: pedometer<br><br>**Duration**:5 days<br><br>**Follow-up post baseline**: 5 days<br><br>**Intro session**: none	**Format:** Facebook app ("StepMatron")<br><br>**Frequency:** daily use encouraged (i.e. total 5 logins)<br><br>**Characteristics**: self-monitoring of daily steps and discussion board/forum<br><br>**Theory**: NR	**Primary outcome:** Objective physical activity<br><br>**Tool:** Pedometer (Silva Ex3 Plus)<br><br>**Validity:** NR	Mean logins = 22 per participant over 21 days.<br><br>Mean 5 page views per visit.<br><br>1min46 sec on step input (non-social) interface; 2min37 on the rankings (social) interface.
Freyne et al, 2010 [23]	CONSORT<br><br>= 3;<br><br>CEBM Level 4	**Participants**: 545 healthy adults with families<br><br>**Age**: NR<br><br>**% female**: NR<br><br>**Participation**	**Focus**: physical activity & diet.<br><br>**Intervention**: online health social network with educational material, self-monitoring, discussion	**Format**: Health online social networking website ("SOFA" – SOcial FAmilies)<br><br>**Frequency**: "Regular use" encouraged	**Primary outcome:** Attitude towards control over health<br><br>**Tool:** Health Locus of Control scale	Social content used more heavily than educational content (5:1 at start, 4:1 by end).<br><br>Website use decreased as the program went on.

Study	Risk of bias (/ 25) and CEBM level of evidence	Design/ participants/ setting	Intervention	Online social-networking features	Outcome measures	Process measures
		**rate**: NR<br><br>**Attrition rate**: NR<br><br>**Recruitment**: NR<br><br>**Setting**: live trial<br><br>**Country**: Australia<br><br>**Design**: single group pre-post	board/forum, quiz<br><br>**Additional to online social network**: nil<br><br>**Duration**:3 weeks<br><br>**Follow-up post baseline**: 3 weeks<br><br>**Intro session**: none	**Characteristics**: education, self-monitoring, discussion board/forum, quiz<br><br>**Theory**: Social Learning Theory	**Validity:** NR	
Kuwata et al, 2010 [29]	CONSORT<br><br>= 4;<br><br>CEBM Level 4	**Participants**: 10 adults with metabolic syndrome<br><br>**Age**: mean 34.4 (SD 6.8)<br><br>**% female**: 40<br><br>**Participation rate**: NR<br><br>**Attrition rate**: 0%<br><br>**Recruitment**: NR<br><br>**Setting**: NR<br><br>**Country**: Japan<br><br>**Design**: single group pre-post	**Focus**: physical activity & diet.<br><br>**Intervention**: Online health social network<br><br>**Additional to online social network**: 7 emails, personalised feedback from dietician plus accelerometer.<br><br>**Duration**: 24 days<br><br>**Follow-up post baseline**: 24 days<br><br>**Intro session**: none	**Format**: Health online social networking website ("Metaboli-net")<br><br>**Frequency**: 3 x daily encouraged (i.e. total 72 logins).<br><br>**Characteristics**: self-monitoring meals, goal-setting, discussion board/forum, facilitator/moderator (dietician)<br><br>**Theory**: NR	**Primary outcome:** Dietary awareness.<br><br>**Tool:** NR<br><br>**Validity:** NR<br><br>**Secondary outcome:** Self-reported physical activity awareness	NR
Ma et al, 2010 [24]	CONSORT<br><br>= 4;<br><br>CEBM Level 4	**Participants**: 107,907<br><br>**Age**: NR	**Focus**: diet, weight loss & physical activity<br><br>**Intervention**: Online health	**Format**: Health online social networking website ("FatSecret")	**Primary outcome:** Self-reported weight<br><br>**Tool:** NA	Mean 6.1 weigh-ins per participant during the 19 week prospective component.

Study	Risk of bias (/ 25) and CEBM level of evidence	Design/ participants/ setting	Intervention	Online social-networking features	Outcome measures	Process measures
		**% female**: NR<br><br>**Participation rate**: NR<br><br>**Attrition rate**: 41%<br><br>**Setting**: live trial<br><br>**Recruitment**: existing users<br><br>**Country**: United States<br><br>**Design**: single group pre-post and retrospective audit	social network<br><br>**Additional to online social network**: fortnightly email reminders<br><br>**Duration**: prospective component – 19 weeks; retrospective component – 24 months<br><br>**Follow-up post baseline**: variable: prospective component – 19 weeks; retrospective component – up to 24 months<br><br>**Intro session**: none	**Frequency**: daily use encouraged<br><br>**Characteristics**: educational content, goal-setting, self-monitoring, discussion board/forum,<br><br>**Theory**: NR	**Validity:** NR	
Napolitano et al, 2012 [28]	CONSORT<br><br>= 11;<br><br>CEBM Level 2	**Participants**: 52 overweight adults<br><br>**Age**: 20.5 (SD 2.2)<br><br>**% female**: 87<br><br>**Participation rate**: 56%<br><br>**Attrition rate**: 4%<br><br>**Recruitment**: advertising within university<br><br>**Setting**: University<br><br>**Country**: United	**Focus**: Physical activity, diet & weight loss<br><br>**Intervention**: (a) Facebook group plus daily SMS messages plus weekly tailored feedback reports; (b) Facebook group; (c) wait-list control.<br><br>**Additional to online social network**: Group (a) 56 SMS messages, pedometer,	**Format**: Facebook group.<br><br>**Frequency**: ? daily<br><br>**Characteristics**: Group (a) Facebook group plus goal-setting, self-monitoring, daily SMS', digital scales, pedometer, book, measuring utensils, personalised weekly report, support buddy.<br><br>Group (b) education, information,	**Primary outcomes:** Objective weight<br><br>**Tool:** scales<br><br>**Validity:** NR<br><br>**Secondary outcomes:** Self-reported physical activity (Godin); exercise goal-setting; physical activity self-efficacy; weight self-efficacy; social support.	Engagement was slightly higher for Group (a) than Group (b).<br><br>Satisfaction was similar for Group (a) and Group (b).

Study	Risk of bias (/ 25) and CEBM level of evidence	Design/ participants/ setting	Intervention	Online social-networking features	Outcome measures	Process measures
		States<br><br>**Design**: RCT (3 groups)	digital scales, extensive written materials, measuring utensils, support buddy plus expert personalised advice. Group (b) nil.<br><br>**Duration**: 8 weeks<br><br>**Follow-up post baseline**: 4 and 8 weeks.<br><br>**Intro session**: yes	polls, discussion board/forum.<br><br>**Theory**: NR		
Sugano & Yamazaki, 2011 [25]	CONSORT<br><br>= 0.5;<br><br>CEBM Level 4	**Participants**: 2500 adults using weight loss website<br><br>**Age**: NR<br><br>**% female**: 100<br><br>**Participation rate**: NR<br><br>**Attrition rate**: NR<br><br>**Setting**: live trial<br><br>**Recruitment**: existing users<br><br>**Country**: Japan<br><br>**Design**: single group pre-post	**Focus**: diet & weight loss<br><br>**Intervention**: Online health social network<br><br>**Additional to online social network**: nil<br><br>**Duration of intervention**: Study 1 > 6 months<br><br>Study 2 > 90 days<br><br>**Follow-up post baseline**: Study 1 > 6 months<br><br>Study 2 > 90 days<br><br>**Intro session**: no	**Format**: Health online social networking website "Yahoo Diet Diary"<br><br>**Frequency**: daily<br><br>**Characteristics**: self-monitoring, discussion board<br><br>**Theory**: NR	**Primary outcome:** Self-reported weight<br><br>**Tool:** NA<br><br>**Validity:** NR<br><br>**Secondary outcomes:** diet diary	More weight loss occurs in the first 90 days of use, than in ensuing 90 day periods.<br><br>Long term use is associated with higher weight loss.<br><br>Higher social interaction is associated with higher weight loss.

Study	Risk of bias (/ 25) and CEBM level of evidence	Design/ participants/ setting	Intervention	Online social-networking features	Outcome measures	Process measures
Turner-McGrievy & Tate, 2011 [27]	CONSORT<br><br>= 19.5;<br><br>CEBM Level 2	**Participants**: 96 overweight adults<br><br>**Age**: Group-a 42.6 (10.7); Group-b: 43.2 (11.7)<br><br>**% female**: 75<br><br>**Participation rate**: 71%<br><br>**Attrition rate**: 10% **Recruitment**: television advertising and mass emails<br><br>**Setting**: community<br><br>**Country**: United States<br><br>**Design**: RCT	**Focus**: physical activity, diet & weight loss<br><br>**Intervention**: (a): podcasts plus 2 x daily tweets plus FatSecret Calorie Counter phone app; (b) podcasts only.<br><br>**Additional to online social network**: expert moderator, 48 podcasts, FatSecret phone app plus extensive written materials.<br><br>**Duration of intervention**: 26 weeks<br><br>**Follow-up post baseline**: 26 weeks<br><br>**Intro session**: yes	**Format**: Twitter posts from facilitator.<br><br>**Frequency**: 2 x daily<br><br>**Characteristics**: education, self-monitoring, discussion board/forum, facilitator, goal-setting, email reminders,<br><br>**Theory**: Social cognitive theory	**Primary outcome:** Objective weight<br><br>**Tool:** scales<br><br>**Validity:** NR<br><br>**Secondary outcomes:** Self-reported physical activity (Paffenbarger); dietary intake; eating behaviours; weight loss self-efficacy	Number of podcast episodes downloaded per group was similar.<br><br>Within Group (a), participants made a mean of 2.1 (SD 3.1) posts to Twitter per week; 94% posted to Twitter at least once. Rate of Twitter posts decreased across the intervention period.
Valle et al, 2013 [26]	CONSORT<br><br>= 18;<br><br>CEBM Level 2	**Participants**: 86 physically inactive, young adult cancer survivors<br><br>**Age**: 31.7 (5.1)<br><br>**% female**: 91<br><br>**Participation rate**: 89%<br><br>**Attrition rate**:	**Focus**: Physical activity<br><br>**Intervention**: (a) Facebook group plus pedometer plus self-monitoring website; (b) Facebook group plus pedometer<br><br>**Additional to online social network**: pedometer,	**Format**: Facebook group + pedometer + self-monitoring website.<br><br>**Frequency**: daily<br><br>**Characteristics**: education, goal-setting, self-monitoring, email reminders, discussion board,	**Primary outcome:** Self-reported physical activity<br><br>**Tool:** Godin<br><br>**Validity:** yes<br><br>**Secondary outcomes:**<br><br>Quality of life, self-reported	Mean number of Facebook posts was similar for Group (a) and (b).<br><br>Group (a) participants set an average of 4.2 + 4.8 goals (supposed to be 12) and completed a mean of 21.9 + 37.9 physical activity entries (supposed to be 84).

Study	Risk of bias (/ 25) and CEBM level of evidence	Design/ participants/ setting	Intervention	Online social-networking features	Outcome measures	Process measures
		23%<br><br>**Recruitment**: online social media campaign, flyers and mass emails<br><br>**Setting**: community<br><br>**Country**: United States<br><br>**Design**: RCT	expert moderator<br><br>**Duration of intervention**: 12 weeks<br><br>**Follow-up post baseline**: 12 weeks<br><br>**Intro session**: introduction e-mail	moderator<br><br>**Theory**: Social cognitive theory	weight.	Usage gradually declined across the 12 weeks.
